# Marital and partnership status and functional and work limitations among U.S. cancer survivors, 1997–2023

**DOI:** 10.1007/s00520-026-11422-2

**Published:** 2026-10-29

**Authors:** Azza Sarfraz, Areesh Mevawalla, Odysseas P. Chatzipanagiotou, Timothy M. Pawlik

**Affiliations:** Department of Surgery, The Ohio State University Wexner Medical Centerand, James Comprehensive Cancer Center , Columbus, OH USA

**Keywords:** Cancer survivorship, Functional limitations, Activity limitations, Marital status, Cohabitation, Employment disruption

## Abstract

**Background:**

Functional limitations (FL) and activity limitations (AL) represent persistent challenges for cancer survivors and are important determinants of long-term health and quality of life. While marital status is a well-recognized social determinant of survival, its role in shaping disability patterns among survivors remains not well defined.

**Methods:**

The National Health Interview Survey (1997–2023), a nationally representative sample of US adults, was used to compare the prevalence of AL and FL among cancer survivors and the general population stratified by marital and partnership status (i.e., married, living with a partner, divorced/separated, widowed, never married). Models were additionally fitted within survivors alone, using married survivors as the reference category. Multivariable survey-weighted logistic and linear regression models were used to estimate adjusted odds ratios (OR) and adjusted mean differences, controlling for sociodemographic and clinical factors.

**Results:**

Among 72,687 cancer survivors and 725,691 adults in the general population, survivors reported a higher weighted prevalence of AL (33.9 vs 14.0%) and FL (61.1 vs 31.3%) (both *p*<0.001). Within survivors, the adjusted prevalence of AL was lowest among married individuals (29.5%, 95% CI 28.8–30.2) and higher among individuals divorced or separated (39.6%, 38.4–40.8), widowed (37.7%, 36.5–38.8), living with a partner (36.9%, 34.1–39.7), and never married (35.3%, 33.4–37.2). In models restricted to survivors with married survivors as the reference, the adjusted odds of AL were elevated among divorced or separated (AOR 1.73, 1.60–1.86), widowed (1.56, 1.46–1.68), cohabiting (1.50, 1.28–1.76), and never-married (1.38, 1.24–1.54) survivors. Divorced or separated survivors had the highest odds of any work limitation (1.53, 1.42–1.65) and of being unable to work (1.56, 1.43–1.71). On multivariable analysis, cancer survivors had 44% higher odds of AL (AOR 1.44, 95% CI 1.39–1.49) and 61% higher odds of FL (AOR 1.61, 1.56–1.66) compared with the general US population. The association between partnership status and AL differed by sex (*p* for interaction=0.005) and by age group (*p*=0.007).

**Conclusions:**

Cancer survivors experience a substantially higher burden of AL and FL than the general US population, with divorced and widowed survivors facing the greatest challenges across physical, social, and vocational domains. Cohabiting survivors resembled unmarried rather than married survivors, indicating that legal marital status alone does not capture the relevant exposure. Survivors without a co-resident spouse as a group had more functional and vocational burden and may warrant prioritization in the design and prospective evaluation of survivorship pathways incorporating functional screening and rehabilitation.

**Supplementary Information:**

The online version contains supplementary material available at https://doi.org/10.1007/s00520-026-11422-2.

## Introduction

Advancements in cancer detection and treatment have led to a United States (US) cancer survivor population exceeding 18 million, a figure projected to reach 26 million by 2040 [1]. Five-year survival in the US has improved markedly, increasing from around 39% in the early 1960 s to approximately 70% by 2020—reflecting major advances in early detection and effective treatments for many cancer types [2]. Despite these gains, functional impairments remain common among survivors and often portend worse clinical outcomes [3]. For example, each additional physical functional limitation, such as difficulty walking or climbing stairs, has been associated with about a 19% higher risk of mortality among cancer survivors [4]. Similarly, in a large cohort of older adults with cancer, individuals who experienced an early functional decline (i.e., loss of at least one activity of daily living [ADL] or instrumental ADL within 3 months of starting treatment) had a 45% increased risk of all-cause mortality [5]. Beyond health outcomes, the consequences of cancer extend into the workplace; an estimated 40–50% of survivors make significant adjustments to their work life due to cancer such as reducing their hours, changing job duties, or taking extended leave [6]. Together, these findings highlight functional decline as a central challenge of survivorship with potential implications for long-term health and quality of life.

While functional limitations are well documented across cancer survivors, growing evidence suggests that nonmarried survivors—particularly individuals who are divorced or widowed—experience a disproportionately greater impact of cancer on functional decline [7]. Some data have suggested that these individuals have higher rates of disability, poorer quality of life, and worse overall survival compared with married counterparts [8, 9]. For example, Chen et al. reported that married individuals had better cancer-specific survival across nine cancer types, a benefit attributed not only to earlier diagnosis and greater treatment receipt but also to preserved functional status through stronger social and instrumental support [10]. Similarly, Krajc et al. noted that unmarried patients experienced worse overall and cancer-specific survival, with divorced/separated men representing the most vulnerable subgroup [9]. In addition to cancer survival, marital status may also influence functional recovery; however, this topic has not been well examined. The present analysis was informed by the marital resource model and by the stress-buffering framework of social relationships, which together propose that partnership confers health advantages through four partially distinct pathways: instrumental support (e.g., transportation to appointments, assistance with household tasks and personal care); economic resources and risk pooling within the household; informal health monitoring and social control of health behavior; and emotional buffering of illness-related stress [11]. In cancer populations specifically, lower perceived social support has been associated with poorer functional recovery and worse health-related quality of life following treatment [12]. Because these pathways operate through the presence of a co-resident partner rather than through legal marriage as such, cohabiting adults were analyzed as a distinct category rather than being combined with either married or never-married respondents [13]. The framework suggests that survivors with a co-resident partner should carry the lowest functional burden and formerly partnered survivors the highest; in addition, the magnitude of any advantage should differ by sex, given consistent evidence that men and women derive health benefits from marriage through different mechanisms.

Therefore, the objective of the current study was to characterize the association of marital and partnership status with physical limitations, reduced ability to maintain independence, and work disruption among adults with a history of cancer. In particular, we sought to characterize the association of marital status as a social determinant in cancer survivorship.

## Methods

### Data source and study population

The National Health Interview Survey (NHIS) is conducted annually by the National Center for Health Statistics (NCHS) and uses a cross-sectional, multistage, probability sampling design to capture a representative sample of the noninstitutionalized civilian population in the United States (US) [14]. The sampling strategy ensures broad demographic and geographic coverage. Data are collected through structured household interviews with one randomly selected adult respondent per family. For the present analysis, NHIS data from 1997 to 2023 were obtained via the Integrated Public Use Microdata Series (IPUMS), a service of the University of Minnesota that harmonizes NHIS data across survey years [15]. Adults aged ≥18 years who self-reported a history of cancer were included in the analytic sample. Individuals with non-melanoma skin cancer were excluded due to the distinct clinical course and lack of systemic therapy requirements compared with other malignancies. As NHIS data are publicly available and de-identified, this study was deemed exempt from institutional review board (IRB) approval. Methodological reporting adhered to the Strengthening the Reporting of Observational Studies in Epidemiology (STROBE) guidelines.

### Covariates and outcomes of interest

Baseline demographic and socioeconomic variables included sex (i.e., male, female), age (i.e., <50, ≥50 years), race (i.e., White, Black, Other), and ethnicity (i.e., Hispanic, non-Hispanic). Additional covariates included body mass index (BMI, i.e., <18.5, 18.5–24.9, 25–29.9, ≥30 kg/m^2^), household income (i.e., below poverty line, low-to-middle income, high income), insurance type (i.e., Medicare, Medicaid, private, uninsured), and employment status (i.e., unemployed, part-time, full-time). The primary exposure of interest was marital and partnership status, derived from the current marital status variable (MARSTCUR) and categorized as married with a spouse in the household, living with a partner, divorced or separated, widowed, or never married. Cohabiting respondents were retained as a distinct category rather than being combined with married or never-married respondents, because NHIS ascertains co-residential partnership directly and collapsing these groups would obscure the exposure of interest. Survivor-specific analyses were restricted to adults with a self-reported history of cancer (Supplementary Table 1), which included breast, prostate, lung, colorectal, melanoma, bladder, non-Hodgkin lymphoma, kidney, leukemia, thyroid, and other cancers

Covariates were specified a priori rather than by data-driven selection and were chosen on the basis of the conceptual framework outlined above together with prior NHIS-based analyses of functional limitation. Eligibility for inclusion required that a variable be plausibly associated with both partnership status and functional outcomes, not lie on the causal pathway between them, and be available and harmonized across all survey years. Age, sex, race, and ethnicity were included as core demographic confounders; household income relative to the federal poverty line and insurance type were included as markers of socioeconomic position and access to care; and body mass index was included as an available marker of general health status. Employment status was included for all outcomes except the three employment outcomes, which lie on the causal pathway between health limitation and work cessation. Several factors (i.e., educational attainment, validated comorbidity indices, sexual orientation, pre-diagnosis functional status, and measures of relationship quality or non-spousal caregiving) that may correlate both partnership status and cancer outcomes could not be included in the analysis due to lack of availability in the analytic extract or not being harmonized across the study period.

The primary outcomes were activity limitations (AL) and functional limitations (FL), assessed using the “LANY” and “FLANY” codes, respectively. Participants were first asked whether they had any limitation due to chronic health condition, with limitations defined as any difficulty in performing a usual activity without special equipment or assistance. Respondents who self-reported “yes” were further asked to specify the health condition(s) responsible for their limitation, including cancer. Although NHIS includes condition-specific attribution variables for selected survey years, these variables were not included in the present analytic extract. Accordingly, the primary AL and FL outcomes were defined as any reported limitation, irrespective of the underlying health condition. These estimates therefore reflect the overall burden of limitations among cancer survivors rather than limitations directly attributable to cancer or its treatment. AL were defined as self-reported difficulty in performing basic daily activities due to a physical, mental, or emotional problem, while FL were defined as self-reported difficulty performing broader physical, cognitive, or social tasks necessary for independent living, as reported previously [16].

Additional outcomes included limitations in activities of daily living (ADLs) and instrumental activities of daily living (IADLs), as well as difficulties with specific physical and social activities, including bathing, dressing, transferring, eating, toileting, prolonged standing or sitting, walking, climbing stairs, leisure activities, and socializing. Self-rated health was assessed on a five-point scale ranging from excellent to poor and dichotomized as poor/fair versus good/very good/excellent. Employment-related outcomes included any work limitation, inability to work, and the number of workdays lost due to illness during the preceding year. Workdays lost was analyzed as a continuous outcome. Detailed NHIS variable names and definitions are provided in Supplementary Table 7.

### Statistical analyses

Descriptive statistics for categorical variables were presented as frequencies and percentages (%) and analyzed using the Chi-square test or Fisher’s exact test, as appropriate. Continuous variables were summarized using means with standard deviations (SD) or medians with interquartile ranges (IQR), as appropriate. Unadjusted prevalence estimates were calculated overall and by marital status among cancer survivors. Covariate-adjusted prevalence (predictive margins) with 95% confidence intervals (CI) were estimated from survey-weighted regression models, stratified by marital status, in both the full US adult sample and the subset of cancer survivors. For binary outcomes, survey-weighted logistic regression models were used; for the continuous outcome (work-lost days), survey-weighted linear regression was used to obtain adjusted means.

NHIS is administered as a series of independent annual cross-section assessments rather than as a longitudinal panel; no respondent was interviewed in more than one survey year. The 1997–2023 waves were therefore pooled and analyzed as repeated cross-sectional data with each respondent contributing a single observation and no within-person clustering. Survey year was retained as a covariate in all multivariable models to account for secular change in the prevalence of reported limitation, in the distribution of partnership status, and in NHIS questionnaire content across the 27-year period, including the 2019 redesign. Variance estimation used Taylor series linearization, with strata and primary sampling units nested within survey year to reflect changes in the NHIS sample design over the study period.

Subsequently, survey-weighted multivariable models compared cancer survivors with the general US population, stratified by marital status. For binary outcomes, adjusted odds ratios (AOR) with 95% CI are reported; for continuous outcomes, β-coefficients represent adjusted mean differences. All multivariable models adjusted for age, sex, race, ethnicity, body mass index, household income, insurance type, employment status (except for the employment outcomes), and survey year. Age was entered as a dichotomous variable (<50 vs ≥50 years) in all multivariable models, consistent with its descriptive categorization; this threshold was selected to separate survivors of working age from those approaching or beyond typical retirement age among whom age-related functional decline becomes substantially more common. To test whether the association between partnership status and limitation differed across subgroups, multiplicative interaction terms (partnership status × sex and partnership status × age group) were fitted in models restricted to cancer survivors and evaluated using adjusted Wald tests. NHIS sampling weights, strata, and primary sampling units were applied in all analyses to account for the complex survey design and to produce nationally representative estimates. Because comparisons against the general population address a different question from the association of partnership status with limitation among survivors, survey-weighted multivariable models were additionally fitted within the cancer survivor sample alone, using married survivors as the reference category and adjusting for the same covariate set (Supplementary Table 6). Respondents with missing values on any model covariate were excluded listwise; the analytic sample for the within-survivor models comprised 40,869 of 72,687 survivors, with exclusions driven principally by missing household income and race.

All statistical tests were two-tailed, and the level of significance was set at *α*=0.05. Statistical analyses were conducted using Stata version 18.0 (StataCorp, College Station, TX).

## Results

### Baseline characteristics

Among 798,378 survey respondents, 72,687 (8.8%) were cancer survivors and 725,691 (91.2%) comprised the general US population. Cancer survivors were comparatively older than the US population (weighted mean age 63.6 ± 14.9 vs 44.9 ± 17.3 years; *p*<0.001). A lower proportion of cancer survivors was male (29,045 [43.1%] vs 325,099 [48.7%]) (*p*<0.001). Compared with the US population, most cancer survivors identified as White (60,339 [90.3%] vs 505,604 [79.1%]) and as non-Hispanic (68,514 [94.9%] vs 604,064 [85.3%]) (both *p*<0.001). Medicare coverage was higher among cancer survivors (43,568 [55.9%] vs 152,325 [17.2%]) and more survivors were not employed (48,414 [63.0%] vs 274,588 [34.8%]) than in the US population (both *p*<0.001). Cancer survivors were more often widowed (16,157 [15.2%] vs 64,437 [5.4%]) and divorced or separated (13,783 [13.4%] vs 118,893 [10.7%]), and less often cohabiting (2280 [4.0%] vs 42,745 [7.3%]) or never married (6386 [6.9%] vs 172,237 [23.0%]) (all *p*<0.001) (Table 1).

**Table 1:** Baseline characteristics of the study cohort

**Characteristics**	**Total (*N*=798,378)**	**US population (*N*=725,691, 91.2%)**	**Cancer survivors (*N*=72,687, 8.8%)**
*N* (unweighted)	798,378	725,691	72,687
Age, mean (SD)	46.4 (17.9)	44.9 (17.3)	63.6 (14.9)
<50 years	426,014 (58.0)	414,959 (61.6)	11,055 (17.6)
≥50 years	372,364 (42.0)	310,732 (38.4)	61,632 (82.4)
Male	354,144 (48.2)	325,099 (48.7)	29,045 (43.1)
Female	444,234 (51.8)	400,592 (51.3)	43,642 (56.9)
Married	361,460 (54.2)	327,379 (53.6)	34,081 (60.6)
Living with partner	45,025 (7.1)	42,745 (7.3)	2280 (4.0)
Divorced/separated	132,676 (10.9)	118,893 (10.7)	13,783 (13.4)
Widowed	80,594 (6.2)	64,437 (5.4)	16,157 (15.2)
Never married	178,623 (21.6)	172,237 (23.0)	6386 (6.9)
White	565,943 (80.1)	505,604 (79.1)	60,339 (90.3)
Black	99,029 (11.9)	93,995 (12.5)	5034 (6.0)
Other	59,388 (8.0)	56,785 (8.4)	2603 (3.7)
Non-Hispanic	672,578 (86.1)	604,064 (85.3)	68,514 (94.9)
Hispanic	125,735 (13.9)	121,565 (14.7)	4170 (5.1)
BMI, mean (SD)	27.4 (6.0)	27.3 (6.0)	27.5 (5.9)
<18.5	12,113 (1.9)	10,852 (1.9)	1261 (2.2)
18.5–24.9	233,524 (36.9)	215,015 (37.1)	18,509 (34.6)
25–29.9	220,245 (34.9)	201,364 (34.8)	18,881 (36.1)
≥30	166,982 (26.3)	152,519 (26.2)	14,463 (27.2)
Below poverty line	103,210 (11.5)	96,171 (11.8)	7039 (8.6)
Low to middle income	334,397 (46.2)	303,537 (46.3)	30,860 (45.2)
High income	265,651 (42.3)	239,013 (41.9)	26,638 (46.2)
Medicare	195,893 (20.4)	152,325 (17.2)	43,568 (55.9)
Medicaid	53,998 (7.0)	51,011 (7.3)	2987 (4.1)
Private	409,628 (58.3)	388,451 (60.4)	21,177 (35.3)
Uninsured	111,594 (14.2)	108,426 (15.1)	3168 (4.7)
Not employed	323,002 (37.1)	274,588 (34.8)	48,414 (63.0)
Part time	99,426 (13.3)	92,397 (13.6)	7029 (10.1)
Full time	375,950 (49.6)	358,706 (51.6)	17,244 (26.8)

*BMI*, body mass index; *kg/m*^*2*^, kilograms per square meter

^*^All *p*-values are <0.001. *N* represents the unweighted number of survey respondents. Weighted percentages are reported

### Cancer-associated limitations

**Fig. 1:**
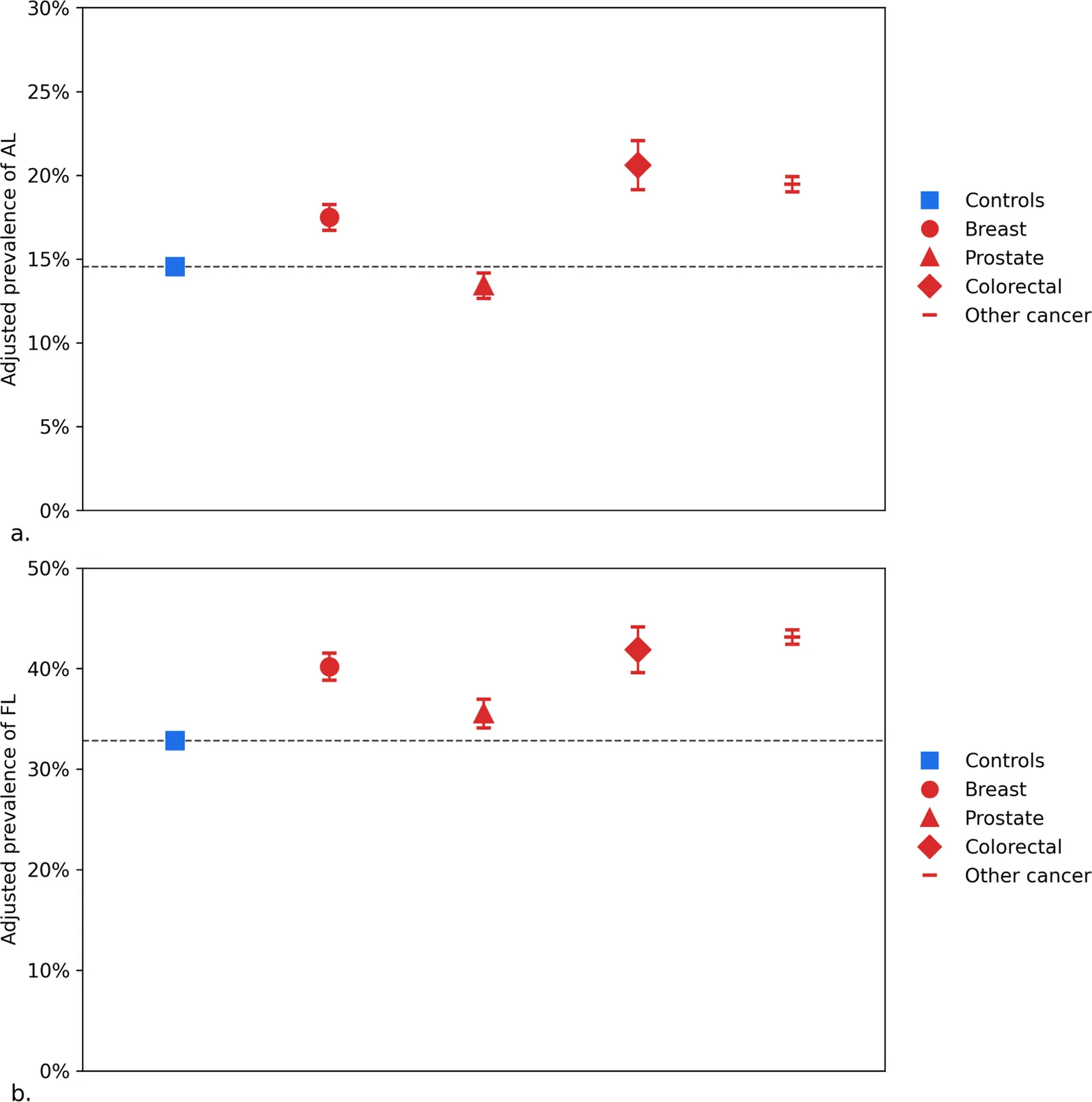
Prevalence of **a** activity limitations (AL) and **b** functional limitations (FL) among cancer survivors by cancer site compared to the general US population. Covariate-adjusted prevalence estimates (predictive margins) from survey-weighted logistic regression models are shown, with error bars representing 95% confidence intervals. The dashed reference line indicates the adjusted prevalence among controls (adults without a cancer history)

Overall, the weighted prevalence of AL and FL was higher among cancer survivors (AL, 33.9 vs 14.0%; FL, 61.1 vs 31.3%) compared with the general US population (both *p*<0.001). Figure 1 illustrates variation in adjusted AL and FL prevalence across cancer types, with colorectal and other cancers demonstrating among the highest estimates. The most reported limitations among cancer survivors were difficulty maintaining standing (40.2 vs 17.3%), climbing stairs (29.9 vs 11.9%), and any work limitation (27.6 vs 12.4%) (all *p*<0.001) (Supplementary Table 2).

Using survey-weighted models across the full cohort, the adjusted prevalence of any AL was lowest among married adults and highest among those divorced or separated and widowed (Table 2). Across all age groups, divorced, separated, or widowed individuals had a higher AL prevalence than married individuals. A comparable gradient was observed for any FL (Table 2). Adjusted FL prevalence increased with age in every partnership category. Poor-to-fair health status was more common among divorced or separated than among never-married individuals (Table 2, Supplementary Table 3).

**Table 2:** Adjusted prevalence of outcomes among cancer survivors and general US population using survey-weighted models, stratified by marital status

**Variables**	**Married**	**Living with partner**	**Divorced/separated**	**Widowed**	**Never married**
Any AL	13.0 (12.8–13.1)	14.9 (14.4–15.4)	20.9 (20.6–21.2)	16.9 (16.6–17.3)	15.6 (15.4–15.9)
Any FL	33.3 (33.1–33.5)	35.5 (34.8–36.1)	38.2 (37.8–38.6)	38.3 (37.7–38.9)	29.3 (29.0–29.7)
Need help with ADL	1.5 (1.5–1.6)	1.7 (1.5–2.0)	2.2 (2.1–2.3)	2.6 (2.5–2.8)	2.3 (2.2–2.5)
Need help with IADL	2.8 (2.7–2.9)	3.1 (2.8–3.4)	5.3 (5.1–5.4)	5.6 (5.4–5.8)	4.8 (4.6–5.0)
Need help with bath/shower	1.0 (0.9–1.1)	1.0 (0.9–1.2)	1.3 (1.2–1.4)	1.9 (1.8–2.0)	1.6 (1.5–1.7)
Need help dressing	1.0 (0.9–1.0)	1.0 (0.8–1.2)	1.0 (0.9–1.1)	1.5 (1.4–1.6)	1.3 (1.2–1.4)
Need help in/out of bed or chairs	0.8 (0.7–0.8)	0.9 (0.7–1.0)	0.9 (0.8–1.0)	1.2 (1.1–1.3)	0.9 (0.8–1.0)
Need help eating	0.3 (0.3–0.3)	0.2 (0.2–0.3)	0.4 (0.3–0.4)	0.6 (0.5–0.6)	0.8 (0.7–0.9)
Need help using the toilet	0.5 (0.5–0.6)	0.4 (0.3–0.6)	0.5 (0.5–0.6)	0.8 (0.8–0.9)	0.8 (0.7–0.9)
Difficulty maintaining standing	18.4 (18.2–18.6)	18.9 (18.3–19.4)	22.2 (21.9–22.5)	22.0 (21.6–22.4)	15.6 (15.3–15.9)
Difficulty maintaining sitting	11.1 (11.0–11.3)	12.2 (11.7–12.6)	14.1 (13.8–14.4)	10.4 (10.1–10.7)	8.0 (7.8–8.2)
Difficulty walking	4.2 (4.1–4.3)	5.1 (4.7–5.5)	6.9 (6.7–7.1)	7.5 (7.3–7.6)	4.9 (4.7–5.1)
Difficulty climbing stairs	11.0 (10.9–11.2)	12.1 (11.6–12.6)	14.5 (14.2–14.7)	14.2 (13.9–14.5)	9.4 (9.1–9.6)
Difficulty relaxing	3.8 (3.7–3.9)	4.1 (3.8–4.4)	5.2 (5.0–5.3)	4.1 (3.9–4.2)	2.9 (2.7–3.0)
Difficulty socializing	6.6 (6.5–6.8)	7.6 (7.2–8.0)	9.7 (9.4–9.9)	8.3 (8.1–8.6)	6.7 (6.5–6.9)
Any work limitation	10.3 (10.1–10.4)	11.4 (11.0–11.9)	16.1 (15.9–16.4)	11.8 (11.5–12.0)	12.9 (12.7–13.2)
Unable to work	6.4 (6.2–6.5)	7.6 (7.2–7.9)	10.5 (10.3–10.7)	6.9 (6.7–7.1)	8.2 (8.0–8.5)
Work-lost days (mean)	3.0 (2.9–3.1)	3.7 (3.5–3.8)	4.0 (3.9–4.1)	3.5 (3.1–3.8)	2.8 (2.8–2.9)
Health status, poor to fair	12.0 (11.8–12.2)	13.1 (12.6–13.6)	15.2 (14.9–15.4)	11.6 (11.4–11.9)	9.9 (9.7–10.2)

*AOR*, adjusted odds ratio; *AL*, activity limitation; *β*, beta-coefficient; *CI*, confidence interval; *FL*, functional limitation

^*^All *p*-values are <0.001

Among cancer survivors, the adjusted prevalence of any AL was lowest in married survivors (29.5% [28.8–30.2%]) and higher among individuals divorced or separated (39.6% [38.4–40.8%]), widowed (37.7% [36.5–38.8%]), living with a partner (36.9% [34.1–39.7%]), and never married (35.3% [33.4–37.2%]). The corresponding prevalence of any FL was 59.7% (59.0–60.5%) in married survivors and was highest among widowed survivors (65.0% [63.7–66.3%]) (all *p*<0.001). The most frequent limitations among divorced or separated survivors were difficulty maintaining standing (41.8% [40.6–43.0%]), any work limitation (30.5% [29.4–31.5%]), and difficulty climbing stairs (27.9% [26.9–29.0%]). Among widowed survivors, the most common were difficulty maintaining standing (44.6% [43.3–45.9%]), difficulty climbing stairs (31.3% [30.2–32.4%]), and any work limitation (26.6% [25.6–27.7%]). Adjusted mean workdays lost in the previous year did not differ materially across partnership categories (Table 3, Supplementary Table 4).

**Table 3:** Adjusted prevalence of outcomes among cancer survivors using survey-weighted models, stratified by marital status

**Variables**	**Married**	**Living with partner**	**Divorced/separated**	**Widowed**	**Never married**
Any AL	29.5 (28.8–30.2)	36.9 (34.1–39.7)	39.6 (38.4–40.8)	37.7 (36.5–38.8)	35.3 (33.4–37.2)
Any FL	59.7 (59.0–60.5)	61.8 (58.9–64.7)	62.2 (61.0–63.4)	65.0 (63.7–66.3)	57.9 (56.0–59.8)
Need help with ADL	4.4 (4.0–4.7)	5.8 (4.3–7.4)	5.0 (4.4–5.5)	6.7 (6.1–7.3)	4.7 (3.7–5.8)
Need help with IADL	7.4 (6.9–7.8)	9.1 (7.3–11.0)	11.6 (10.8–12.4)	13.9 (13.2–14.7)	11.0 (9.6–12.4)
Need help with bath/shower	2.9 (2.7–3.2)	4.4 (3.1–5.8)	2.8 (2.4–3.3)	5.1 (4.6–5.6)	3.0 (2.2–3.9)
Need help dressing	2.5 (2.2–2.8)	3.4 (2.2–4.6)	2.2 (1.8–2.6)	3.9 (3.4–4.4)	2.2 (1.5–3.0)
Need help in/out of bed or chairs	2.1 (1.9–2.4)	2.9 (1.8–4.0)	2.0 (1.6–2.3)	2.8 (2.4–3.2)	1.8 (1.1–2.5)
Need help eating	0.8 (0.7–1.0)	1.1 (0.4–1.8)	1.0 (0.7–1.2)	1.4 (1.1–1.7)	1.0 (0.4–1.7)
Need help using the toilet	1.5 (1.3–1.7)	1.4 (0.6–2.2)	1.2 (0.9–1.5)	2.2 (1.8–2.6)	1.0 (0.6–1.4)
Difficulty maintaining standing	38.1 (37.4–38.9)	40.7 (37.8–43.6)	41.8 (40.6–43.0)	44.6 (43.3–45.9)	37.6 (35.7–39.6)
Difficulty maintaining sitting	18.5 (17.9–19.2)	21.9 (19.4–24.5)	21.6 (20.6–22.6)	17.9 (16.9–18.9)	16.8 (15.3–18.3)
Difficulty walking	11.0 (10.5–11.5)	14.9 (12.4–17.3)	15.6 (14.7–16.5)	19.3 (18.5–20.2)	15.1 (13.5–16.7)
Difficulty climbing stairs	24.3 (23.6–24.9)	28.4 (25.6–31.1)	27.9 (26.9–29.0)	31.3 (30.2–32.4)	24.5 (22.7–26.2)
Difficulty relaxing	7.0 (6.6–7.5)	9.1 (7.3–10.9)	8.6 (7.9–9.3)	8.1 (7.4–8.7)	5.7 (4.8–6.7)
Difficulty socializing	14.7 (14.1–15.2)	16.6 (14.2–19.0)	18.4 (17.4–19.4)	18.0 (17.1–19.0)	15.1 (13.5–16.6)
Any work limitation	23.2 (22.5–23.8)	28.5 (25.8–31.2)	30.5 (29.4–31.5)	26.6 (25.6–27.7)	27.3 (25.5–29.1)
Unable to work	14.2 (13.6–14.7)	17.6 (15.3–20.0)	19.7 (18.8–20.6)	16.2 (15.4–17.1)	17.0 (15.5–18.5)
Work-lost days (mean)	5.7 (5.3–6.0)	6.0 (4.7–7.3)	6.3 (5.7–6.8)	6.3 (5.1–7.4)	5.8 (5.1–6.6)
Health status, poor to fair	24.6 (23.9–25.3)	27.7 (25.1–30.2)	27.8 (26.7–28.8)	23.5 (22.5–24.5)	23.2 (21.5–24.9)

^*^All *p*-values are <0.001

**Fig. 2:**
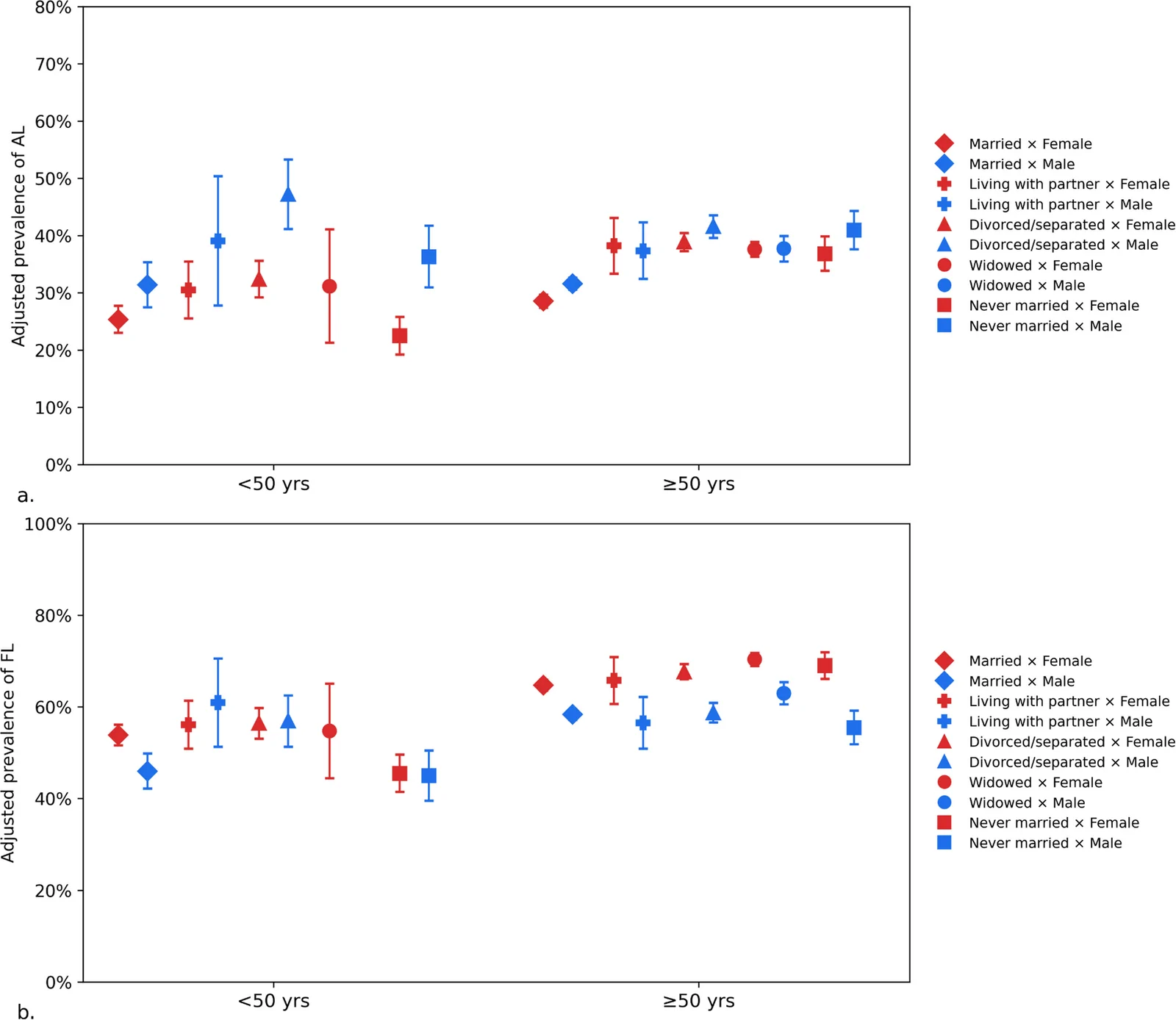
Adjusted prevalence of **a** activity limitations (AL) and **b** functional limitations (FL) among cancer survivors only, stratified by marital and partnership status, sex, and age group (<50 vs ≥50 years). Estimates are covariate-adjusted predictive margins from survey-weighted logistic regression models; error bars represent 95% confidence intervals. Female estimates are shown in red and male estimates in blue. Estimates for widowed men aged <50 years are not displayed because only 17 survivors fell into this cell

Among cancer survivors, the adjusted prevalence of AL and FL varied by both sex and age within partnership categories (Fig. 2). For AL (Fig. 2a), male survivors reported a higher adjusted prevalence than female survivors in every partnership category among individuals aged <50 years; the difference was widest among divorced or separated survivors (47.2% [41.2–53.3%] in men vs 32.4% [29.2–35.6%] in women) and never-married survivors (36.3 [30.9–41.8%] vs 22.5% [19.2–25.8%]) compared with a difference of approximately 6 percentage points among married survivors of the same age (31.4 vs 25.4%). Among survivors aged ≥50 years, these differences narrowed substantially, and male and female estimates converged within each category. For FL (Fig. 2b), female survivors reported a higher adjusted prevalence than male survivors in most categories, most notably among individuals aged ≥50 years who were widowed (70.4 [69.0–71.8%] vs 63.0% [60.6–65.4%]) or never married (69.0 [66.1–72.0%] vs 55.5% [51.9–59.2%]). Formal tests of interaction indicated that the association of partnership status with AL differed by sex (*p* for interaction=0.005) and by age (*p*=0.007). For FL, the interaction with age was significant (*p*=0.002) whereas the interaction with sex was not (*p*=0.93), indicating that the higher FL prevalence among women reflected a main effect of sex rather than a partnership-specific pattern. There were only 17 survivor widowed men aged <50 years preventing any subset analysis in this cohort.

### Multivariable analyses

After controlling for competing risk factors on multivariable logistic regression, cancer survivors had 44% higher odds of AL (AOR 1.44 [95% CI 1.39–1.49], *p*<0.001) compared with the general US population. Similar trends were observed for FL, with cancer survivors having 61% higher odds (AOR 1.61 [1.56–1.66], *p*<0.001) (Supplementary Table 5). In models stratified by partnership status that used the general US population as a reference, cancer survivors had higher odds of any AL within every partnership category (Table 4). The same was true for any FL within every partnership category (Table 4).

**Table 4:** Multivariable analysis of outcomes cancer survivors compared to US population using survey-weighted models, stratified by marital status

**Variables (Ref: US population)**	**Married**	**Living with partner**	**Divorced/separated**	**Widowed**	**Never married**
Any AL	1.51 (1.44–1.59)	2.23 (1.85–2.68)	1.41 (1.31–1.52)	1.33 (1.25–1.42)	1.57 (1.36–1.80)
Any FL	1.59 (1.53–1.66)	1.87 (1.61–2.16)	1.57 (1.47–1.68)	1.43 (1.34–1.54)	1.96 (1.76–2.18)
Need help with ADL	1.42 (1.27–1.59)	2.29 (1.58–3.31)	1.31 (1.13–1.51)	1.22 (1.10–1.36)	0.94 (0.73–1.21)
Need help with IADL	1.45 (1.32–1.58)	1.91 (1.43–2.54)	1.28 (1.16–1.42)	1.24 (1.15–1.33)	1.12 (0.93–1.35)
Need help with bath/shower	1.44 (1.26–1.65)	2.88 (1.82–4.55)	1.21 (1.01–1.46)	1.23 (1.09–1.38)	0.87 (0.63–1.20)
Need help dressing	1.28 (1.12–1.47)	2.22 (1.36–3.61)	1.20 (0.97–1.48)	1.21 (1.05–1.38)	0.78 (0.55–1.10)
Need help in/out of bed or chairs	1.33 (1.14–1.55)	2.17 (1.33–3.54)	1.36 (1.09–1.69)	1.15 (0.98–1.36)	0.91 (0.61–1.34)
Need help eating	1.32 (1.04–1.68)	4.77 (1.93–11.79)	1.53 (1.09–2.14)	1.06 (0.86–1.31)	0.61 (0.34–1.09)
Need help using the toilet	1.37 (1.14–1.65)	1.72 (0.79–3.74)	1.32 (0.99–1.77)	1.17 (0.96–1.42)	0.53 (0.35–0.79)
Difficulty maintaining standing	1.48 (1.42–1.55)	1.78 (1.52–2.09)	1.41 (1.31–1.51)	1.34 (1.26–1.42)	1.76 (1.56–1.98)
Difficulty maintaining sitting	1.24 (1.18–1.31)	1.68 (1.42–1.99)	1.22 (1.14–1.32)	1.14 (1.06–1.23)	1.57 (1.38–1.78)
Difficulty walking	1.30 (1.21–1.39)	1.66 (1.28–2.16)	1.18 (1.08–1.28)	1.26 (1.17–1.34)	1.36 (1.16–1.60)
Difficulty climbing stairs	1.40 (1.33–1.48)	1.80 (1.50–2.16)	1.27 (1.18–1.37)	1.29 (1.21–1.37)	1.65 (1.44–1.88)
Difficulty relaxing	1.21 (1.11–1.32)	1.87 (1.47–2.38)	1.22 (1.10–1.35)	1.21 (1.10–1.34)	1.29 (1.06–1.56)
Difficulty socializing	1.38 (1.30–1.47)	1.61 (1.31–1.98)	1.34 (1.23–1.46)	1.23 (1.14–1.32)	1.38 (1.19–1.60)
Any work limitation	1.54 (1.46–1.62)	2.16 (1.77–2.63)	1.42 (1.33–1.53)	1.28 (1.20–1.36)	1.47 (1.28–1.69)
Unable to work	1.41 (1.32–1.51)	1.70 (1.36–2.13)	1.30 (1.20–1.40)	1.26 (1.17–1.36)	1.26 (1.08–1.47)
Work-lost days (mean)	2.55 (2.14–2.96)	2.74 (1.36–4.12)	2.67 (2.03–3.30)	2.43 (1.42–3.44)	3.63 (2.68–4.58)
Health status, poor to fair	1.63 (1.55–1.72)	2.00 (1.69–2.36)	1.58 (1.47–1.70)	1.34 (1.25–1.43)	2.07 (1.82–2.36)

*ADL*, activities of daily living; *AOR*, adjusted odds ratio; *AL*, activity limitation; *β*, beta-coefficient; *CI*, confidence interval; *FL*, functional limitation; *IADL*, instrumental activities of daily living; *ref*, reference

^*^AOR and β-coefficients represent estimates from survey-weighted regression models. Odds ratios are reported for binary outcomes and β-coefficients represent mean differences for continuous outcomes. 95% confidence intervals are reported

In models restricted to cancer survivors with married survivors as the reference category, partnership status remained independently associated with limitation after adjustment. The adjusted odds of any AL were elevated among survivors who were divorced or separated (AOR 1.73, 95% CI 1.60–1.86), widowed (1.56, 1.46–1.68), living with a partner (1.50, 1.28–1.76), and never married (1.38, 1.24–1.54). For any FL, the odds were elevated among widowed (1.31, 1.21–1.41) and divorced or separated (1.13, 1.05–1.22) survivors, whereas cohabiting (1.11, 0.95–1.29) and never-married (0.91, 0.83–1.01) survivors did not differ from married survivors (all *p*>0.05). A comparable gradient was observed for the need for assistance with instrumental activities of daily living (widowed 2.17, 1.96–2.40; divorced or separated 1.72, 1.53–1.92; never married 1.61, 1.34–1.92), for difficulty walking (widowed 2.12, 1.93–2.31), for any work limitation (divorced or separated 1.53, 1.42–1.65), and for inability to work (divorced or separated 1.56, 1.43–1.71). Across nearly every outcome, cohabiting survivors resembled unmarried survivors rather than married survivors (Supplementary Table 6).

## Discussion

Over the recent decades, major advances in early detection and treatment have led to a growing population of cancer survivors with an estimated 18.6 million in the US as of 2025 [17]. However, survivorship care has lagged as many survivors contend with enduring physical, cognitive, and psychosocial sequelae that constrain mobility, self-care, work, and social participation; in addition, rehabilitation and supportive services remain inconsistently integrated into routine oncology care [18]. These functional burdens are shaped not only by tumor and treatment factors but also by social context [19]. Marital status, a pragmatic marker of household support and caregiving, may influence adherence to rehabilitation, access to instrumental help, and resilience in the face of work disruption, yet population-level estimates of cancer-related disability across marital strata are limited [20]. To address this gap, the current study leveraged nationally representative NHIS data to quantify the burden of any AL and any FL among adults with and without a history of cancer, as well as examine how these outcomes differ across married, never married, divorced, and widowed groups. Of note, a wide range of domains including daily functioning, work disruption, and self-rated health were examined. Of note, nearly one-third of US cancer survivors reported at least one AL attributable to a health problem, over twice the prevalence observed among adults without a cancer history. Likewise, about two-thirds of survivors reported FL, which was almost double the prevalence in the general population. Partnership status was associated with functional burden, whereby divorced and widowed cancer survivors consistently demonstrated the highest adjusted prevalence of any AL and FL; in contrast, married counterparts had the lowest, with never-married individuals falling in between. These gradients persisted across multiple life domains (e.g., work limitation, IADL/ADL assistance), highlighting the potential buffering role of spousal support in survivorship [21].

Prior research has estimated that up to 70% of cancer survivors suffer long-term difficulties with mobility, cognition, or daily activities arising from the disease and its treatment, yet these disability issues are not prioritized in cancer care [3]. A recent investigation by Rodin and colleagues indicated that widowhood precipitated clear functional deterioration in older cancer adults with an immediate 1.17-point decline on an 11-point ADL/IADL independence scale, roughly equivalent to losing independence in one daily task [8]. Data from the present study add to previous evidence and identify partnership status as a consistent marker of survivorship disability burden with certain functional domains most impacted by cancer within and across marital strata. Across this nationally representative sample, married survivors had the lowest adjusted prevalence of AL, whereas widowed survivors had the highest adjusted prevalence of FL. Among divorced adults, cancer survivors were most likely to report limitations in standing or sitting for prolonged periods, socializing, and going to work, as well as worse overall health status. Similarly, widowed cancer survivors reported difficulties in standing, climbing stairs, going to work, maintaining employment, and socializing. Previous population-level data on non-married older cancer survivors reported by Westrick et al. identified distinct functional decline phenotypes with widowed survivors in the poorest trajectories compared with married/partnered survivors [22]. Previous studies provided little insight, however, into the types of functional limitations survivors experience. In the current study, consistently observed limitations among divorced/widowed cancer survivors included those related to mobility (i.e., walking, climbing stairs), lower-extremity function (i.e., sitting, standing for prolonged periods), social participation (i.e., socializing), and employment (i.e., going to work, disruption, workdays lost). These data are consistent with the presence of a co-resident partner functioning as a pragmatic marker of available household support. The cross-sectional design cannot, however, establish whether such support mitigated functional deterioration, whether functional status itself influenced the likelihood of entering or remaining in a partnership, or whether both reflected an unmeasured common cause.

Many cancer survivors, particularly those of working age, experience significant work-related disruptions due to cancer and its treatment. National surveys indicate that roughly 74% of working survivors need to miss work during therapy, and more than 1 in 10 ultimately leave or lose their jobs as a result [23]. Likewise, about 40–50% of survivors report making work adjustments (i.e., reduced hours, altered duties, or extended leave) because of cancer [24]. These employment disruptions impose a substantial economic burden; the annual productivity loss attributable to cancer among working-age Americans is estimated at $9.6–$16 billion [25]. Importantly, these vocational consequences are closely tied to survivors’ functional health; individuals with greater physical or cognitive limitations are not only more likely to exit the workforce but also face worse long-term clinical outcomes [26]. In the present study, divorced or separated survivors had the highest adjusted odds of any work limitation and of being unable to work, demonstrating a greater vocational burden within this group. Adjusted workdays lost did not differ materially across partnership categories, and the employment findings were based on the capacity-based measures rather than on days absent. In addition, functional decline often leads to time away from work or job loss, which worsens financial hardship [27]. An estimated 41% of cancer survivors require extended sick leave, reduced hours, or early retirement, leading to lost income and benefits. Policies that support flexible medical leave, enforce anti-discrimination protections, and provide job retraining programs are needed to reduce these burdens [28]. Cancer survivors without a spouse or life-long partner may be particularly at risk for work loss and financial harm.

An important interpretive distinction concerns the direction of the gradient across the two sets of models. Among survivors, the adjusted prevalence of AL and FL was lowest among married individuals and highest among individuals divorced, separated, or widowed. Comparing survivors with the general population within each partnership category, the adjusted odds ratios was lower—perhaps because adults who are divorced, separated, or widowed in the general population already carried a substantially elevated burden of limitation, which compresses the incremental contribution attributable to a cancer history in those strata. The stratified comparison against the general population quantified how much a cancer history added within each partnership group, whereas the within-survivor comparison quantified how functional burden was distributed across partnership groups among survivors.

A distinct contribution of the present analysis is the separation of cohabiting survivors from both married and never-married groups. Cohabitation has become substantially more common in the US across the study period as rates of legal marriage have declined [29]. In turn, analyses that rely solely on legal marital status alone may be inadequate and misleading as cohabiting survivors can be classified as unmarried rather than married survivors. Such a classification may lead to inappropriate inferences related to the impact of cohabitation on any AL, any work limitation, and inability to work. Interestingly, in the current study, the functional advantage observed for married survivors did not appear to be conferred by co-residence with a partner alone. Studies that combine cohabiting with married respondents may attenuate differences between partnered and unpartnered survivors, while including survivors who are cohabitating with never-married respondents will attenuate the effect in the opposite direction. Neither convention is neutral, and reporting cohabitation separately is an important consideration. In addition, the marital resource model may require supplementation by factors such as relationship duration, legal and financial entitlements, or selection into marriage.

Sex-specific patterns merit attention given potential differential health returns related to marriage for men and women. Men derive larger health benefits from being married, whereas women’s health is more sensitive to marital quality than to marital status itself, in part because women more often provide health monitoring and caregiving within the household rather than receiving it [30]. Population-level analyses have likewise reported that the morbidity and mortality penalty associated with marital dissolution is larger for men [30]. The current data were partially consistent with these findings. Among survivors aged <50 years, the excess adjusted prevalence of AL associated with being unpartnered was considerably larger for men than for women, and the interaction between partnership status and sex was notable for AL. Although female survivors reported a higher prevalence of FL overall, this finding reflected a main effect of sex rather than a sex-specific marital advantage. The marital advantage in survivorship therefore appeared to be sex-dependent for activity limitation but not for functional limitation, a distinction that has not previously been noted yet suggests caution against treating the two constructs interchangeably.

Functional status has also emerged as an important prognostic marker in survivorship [31]. Functional impairments are common and portend worse outcomes; for example, each additional physical functional limitation (e.g., difficulty walking or climbing steps) has been associated with about a 19% higher mortality risk in survivors [4]. Similarly, in a large cohort of older adults with cancer, individuals who experienced early functional decline (i.e., loss of ≥1 ADL/IADL within 3 months of treatment) had a 45% increased risk of all-cause mortality [5]. These findings underscore that mitigating the employment impact and preserving functional health are critical in cancer survivorship, as these factors may improve quality of life and potentially enhance long-term survival outcomes [32]. Findings from the current study and prior research highlight an urgent need to incorporate management of functionality into routine survivorship care [33]. If unmanaged, impairments such as weakness, balance issues, or memory difficulties may worsen over time, limiting survivors’ ability to care for themselves, work, or participate in family and community life [34]. Early rehabilitation programs can help patients maintain daily activities and independence [35]. Access to mental health services and peer support is also critical to address the emotional and psychological challenges of living with cancer-related disability, especially for vulnerable groups such as unmarried survivors [36]. These efforts should be specifically targeted to high-risk cancer survivors, such as individuals without a spouse or life-long partner, who lack social support and have the highest adjusted prevalence of any AL and any FL.

The cohort used in the currently analysis was a nationally representative sample that leveraged standardized NHIS measures to examine a broad spectrum of functioning with the systematic stratification by marital status. Despite these strengths, limitations inherent to NHIS included reliance on self-reported cancer history and self-reported limitations, introducing possible recall and reporting bias that may have misestimated prevalence. Furthermore, data from NHIS did not separate limitations due to cancer versus treatment effects or comorbid conditions. In addition, several clinical details (i.e., stage, treatment modality/intensity, recurrence, time since diagnosis) were unavailable, precluding stratification by disease trajectory and limiting causal interpretation. Also, marital status was assessed as a cross-sectional variable and did not capture duration, timing of transitions relative to diagnosis, relationship quality, or non-spousal caregiving; residual confounding by social support may be present, and reverse causation remained a possibility. In addition, despite survey weighting and covariate adjustment, unmeasured factors (e.g., wealth, workplace accommodations, mental health) may have confounded the results.

Certain limitations warrant emphasis. First, marital status is a proxy for the availability of household support rather than a measure of it, and the mechanisms invoked here, instrumental assistance, economic pooling, health monitoring, and emotional buffering, were not directly observed. Second, educational attainment was not available in the analytic extract and could not be included as a covariate. Education is collected in NHIS throughout the study period and is a recognized correlate of both partnership status and functional outcomes independent of income; although household income relative to the federal poverty line and insurance type were included as markers of socioeconomic position, residual confounding by education cannot be excluded and this should be regarded as a substantive limitation rather than a technical one. Third, a validated comorbidity index could not be constructed because the NHIS chronic condition modules were not harmonized consistently across the full study period; self-rated health was therefore analyzed as an outcome rather than as an adjustment variable, and some of the excess limitation observed among survivors may reflect accumulated non-cancer chronic disease, which is itself patterned by partnership status. Fourth, the study period spanned a fundamental change in the legal status of same-sex marriage in the US, which was recognized nationwide in 2015 following Obergefell v. Hodges and in a subset of states before that date. Adults in same-sex partnerships who were living as married during much of the earlier period could not have been legally married; most would have been captured in the cohabiting category used here, which mitigates but does not eliminate the resulting misclassification. Sexual orientation is not consistently available across the full NHIS series and could not be included as a covariate, so the extent of this misclassification could not be quantified. Finally, listwise deletion excluded 31,818 of 72,687 survivors (43.8%) from the within-survivor models, principally because of missing household income and race. Excluded respondents were older and more likely to be widowed than those retained, so the reported estimates may understate the burden concentrated in formerly partnered survivors.

In conclusion, US cancer survivors carried a markedly higher burden of activity and functional limitations than adults without a cancer history. Among survivors, functional outcomes were associated with partnership status. Married survivors had the lowest adjusted prevalence of any AL and any FL and divorced, separated, and widowed survivors the highest; cohabiting survivors were more similar to unmarried rather than married survivors. These disparities extended to work disruption and the need for assistance with daily activities, underscoring the importance of integrating functional screening, rehabilitation, and targeted psychosocial support into survivorship care, particularly for unmarried survivors. Given the descriptive and cross-sectional nature of the data, the current study did not establish causation between functional outcomes and marital status, nor did we identify interventions that might reduce observed disparities. The data do suggest, however, that survivors without a co-resident spouse as a group had more functional and vocational burden and may warrant prioritization in the design and prospective evaluation of survivorship pathways incorporating functional screening and rehabilitation.

## Supplementary Information

Below is the link to the electronic supplementary material.

ESM 1 (DOCX 31.2 KB)

## Data Availability

The data for this study were obtained from the Integrated Public Use Microdata Series National Health Interview Survey (IPUMS NHIS), which is publicly available.

## References

[1] Ehlers DK, Weaver N, Ma J, et al. (2024) Functional limitations among adult cancer survivors in the United States. J Cancer Surviv. Published online 1-11. 10.1007/s11764-024-01638-8 PMC12257919 39083112

[2] American Cancer Society. Cancer Facts & Figures 2025. Published online 2025. Accessed August 22, 2025. https://www.cancer.org/research/cancer-facts-statistics/all-cancer-facts-figures/2025-cancer-facts-figures.html

[3] Nekhlyudov L, Campbell GB, Schmitz KH et al (2021) Cancer‐related impairments and functional limitations among long‐term cancer survivors: gaps and opportunities for clinical practice. Cancer 128(2):222 10.1002/cncr.33913 PMC9292035 34529268

[4] Brown JC, Harhay MO, Harhay MN (2016) Patient-reported versus objectively-measured physical function and mortality risk among cancer survivors. J Geriatr Oncol 7(2):108–115 10.1016/j.jgo.2016.01.009 PMC4818688 26907563

[5] Giri S, Griffin R, Newbrough M et al (2025) Risk of early functional decline and its association with overall survival among older adults with newly diagnosed gastrointestinal malignancies undergoing systemic therapy. J Geriatr Oncol 16(7):102323 10.1016/j.jgo.2025.102323 40795782

[6] de Moor JS, Kent EE, McNeel TS et al (2021) Employment outcomes among cancer survivors in the United States: implications for cancer care delivery. JNCI J Natl Cancer Inst 113(5):641–644 10.1093/jnci/djaa084 PMC7745836 32533839

[7] Aizer AA, Chen MH, McCarthy EP et al (2013) Marital status and survival in patients with cancer. J Clin Oncol 31(31):3869–3876 10.1200/JCO.2013.49.6489 PMC4878087 24062405

[8] Rodin R, Smith AK, Espejo E et al (2024) Mortality and function after widowhood among older adults with dementia, cancer, or organ failure. JAMA Netw Open 7(9):e2432979 10.1001/jamanetworkopen.2024.32979 PMC11393717 39264625

[9] Krajc K, Miroševič Š, Sajovic J et al (2023) Marital status and survival in cancer patients: a systematic review and meta‐analysis. Cancer Med 12(2):1685–1708 10.1002/cam4.5003 PMC9883406 35789072

[10] Chen Z, Yin K, Zheng D et al (2020) Marital status independently predicts non-small cell lung cancer survival: a propensity-adjusted SEER database analysis. J Cancer Res Clin Oncol 146(1):67–74 10.1007/s00432-019-03084-x PMC11804430 31786738

[11] Evans A, Gray E, Reimondos A (2023) Having a partner or living with a partner: differences in life satisfaction and mental health. Appl Res Qual Life 18(5):2295–2313. 10.1007/s11482-023-10186-9

[12] Cho H, Kang D, Shin DW et al (2024) Social support during re-entry period and long-term quality of life in breast cancer survivors: a 10-year longitudinal cohort study. Qual Life Res 33(5):1287–1295. 10.1007/s11136-024-03599-y 38321193

[13] Evans A, Gray E, Reimondos A (2023) Having a partner or living with a partner: differences in life satisfaction and mental health. Appl Res Qual Life 18(5):2295–2313. 10.1007/s11482-023-10186-9

[14] National Center for Health Statistics. Accessed February 10, 2025. https://www.cdc.gov/nchs/nhis/index.htm

[15] Blewett LA, Rivera Drew JA, King ML, Williams KC, Del Ponte N, Convey P (2019) IPUMS health surveys: National Health Interview Survey. IPUMS. Published online, Minneapolis, MN

[16] Sarfraz A, Altaf A, Khalil M et al (2026) Prevalence and trends of cancer-related daily life limitations among gastrointestinal cancer survivors. J Cancer Surviv. 10.1007/s11764-025-01833-1 PMC13676644 40411677

[17] Wagle NS, Nogueira L, Devasia TP et al (2025) Cancer treatment and survivorship statistics, 2025. A Cancer Journal for Clinicians. Published online, CA 10.3322/caac.70011 PMC12223361 40445120

[18] Zebrack B (2024) Cancer survivorship—a framework for quality cancer care. J Natl Cancer Inst 116(3):352–355 10.1093/jnci/djad266 38113418

[19] Gomez SL, Shariff‐Marco S, DeRouen M et al (2015) The impact of neighborhood social and built environment factors across the cancer continuum: current research, methodological considerations, and future directions. Cancer 121(14):2314–2330 10.1002/cncr.29345 PMC4490083 25847484

[20] Yang IS, Rosenfield D, Bowen HJ, Wilson SJ (2026) Daily associations between caregiving time and spousal caregivers’ well-being: the moderating roles of daily marital interactions. Am J Geriatr Psychiatry 34(1):18–27. 10.1016/j.jagp.2025.08.011 41006107

[21] Litzelman K, Yabroff KR (2015) How are spousal depressed mood, distress, and quality of life associated with risk of depressed mood in cancer survivors? Longitudinal findings from a national sample. Cancer Epidemiol Biomarkers Prev 24(6):969–977 10.1158/1055-9965.EPI-14-1420 PMC4453017 26033755

[22] Westrick AC, Langa KM, Eastman M, Ospina-Romero M, Mullins MA, Kobayashi LC (2023) Functional aging trajectories of older cancer survivors: a latent growth analysis of the US Health and Retirement Study. J Cancer Surviv 17(5):1499–1509 10.1007/s11764-022-01185-0 PMC9411262 35218520

[23] American Cancer Society Cancer Action Network. Survivor views: missed work and paid leave. Published online December 2021. Accessed August 22, 2025. http://www.fightcancer.org/survivor-views

[24] Ekwueme DU, Yabroff KR, Guy GP Jr et al (2014) Medical costs and productivity losses of cancer survivors—United States, 2008–2011. MMWR Morb Mortal Wkly Rep. 63(23):505–510 PMC5779368 24918485

[25] Guy GP Jr, Ekwueme DU, Yabroff KR et al (2013) Economic burden of cancer survivorship among adults in the United States. J Clin Oncol 31(30):3749–3757 10.1200/JCO.2013.49.1241 PMC3795887 24043731

[26] Mehnert A, de Boer A, Feuerstein M (2013) Employment challenges for cancer survivors. Cancer 119:2151–2159 10.1002/cncr.28067 23695927

[27] Blinder VS, Gany FM (2019) Impact of cancer on employment. J Clin Oncol 38(4):302 10.1200/JCO.19.01856 PMC6992498 31804857

[28] de Moor JS, Williams CP, Blinder VS (2022) Cancer-related care costs and employment disruption: recommendations to reduce patient economic burden as part of cancer care delivery. JNCI Monogr 2022(59):79–84 10.1093/jncimonographs/lgac006 PMC9255940 35788373

[29] Lundberg S, Pollak RA, Stearns J (2016) Family inequality: diverging patterns in marriage, cohabitation, and childbearing. J Econ Perspect 30(2):79–102. 10.1257/jep.30.2.79 PMC4861075 27170828

[30] Wang Y, Jiao Y, Nie J et al (2020) Sex differences in the association between marital status and the risk of cardiovascular, cancer, and all-cause mortality: a systematic review and meta-analysis of 7,881,040 individuals. Glob Health Res Policy 5:4. 10.1186/s41256-020-00133-8 PMC7047380 32161813

[31] Petrick JL, Reeve BB, Kucharska-Newton AM et al (2014) Functional status declines among cancer survivors: trajectory and contributing factors. J Geriatr Oncol 5(4):359–367 10.1016/j.jgo.2014.06.002 PMC4254190 24981125

[32] Lustberg MB, Kuderer NM, Desai A, Bergerot C, Lyman GH (2023) Mitigating long-term and delayed adverse events associated with cancer treatment: implications for survivorship. Nat Rev Clin Oncol 20(8):527–542 10.1038/s41571-023-00776-9 PMC10211308 37231127

[33] Emery J, Butow P, Lai-Kwon J, Nekhlyudov L, Rynderman M, Jefford M (2022) Management of common clinical problems experienced by survivors of cancer. Lancet 399(10334):1537–1550 10.1016/S0140-6736(22)00242-2 35430021

[34] Mayer RS, Engle J (2022) Rehabilitation of individuals with cancer. Ann Rehabil Med 46(2):60–70 10.5535/arm.22036 PMC9081390 35508925

[35] Martínez-Velilla N, Cadore E, Casas-Herrero Á, Idoate-Saralegui F, Izquierdo M (2016) Physical activity and early rehabilitation in hospitalized elderly medical patients: systematic review of randomized clinical trials. J Nutr Health Aging 20(7):738–751 10.1007/s12603-016-0683-4 PMC12879149 27499308

[36] Joshy G, Thandrayen J, Koczwara B et al (2020) Disability, psychological distress and quality of life in relation to cancer diagnosis and cancer type: population-based Australian study of 22,505 cancer survivors and 244,000 people without cancer. BMC Med 18(1):372 10.1186/s12916-020-01830-4 PMC7708114 33256726

