# Supplementary material for: Marital and partnership status and functional and work limitations among U.S. cancer survivors, 1997–2023
Source: Support Care Cancer. 2026 Oct 29;34(11):1168. doi: 10.1007/s00520-026-11422-2 (PMC13719778; doi:10.1007/s00520-026-11422-2)
Supplement: Supplementary file 1 [file 520_2026_11422_MOESM1_ESM.docx]

**Supplementary Table 1.** Cancer Type Distribution Among Cancer Survivors in the Study Cohort.

| **Characteristic** | **Cancer Survivors**  **(n = 72,687)** |
| --- | --- |
| Breast | 13,677 (18.4%) |
| Prostate | 8,315 (10.9%) |
| Lung | 2,001 (2.7%) |
| Colorectal | 4,497 (6.5%) |
| Melanoma | 4,072 (6.2%) |
| Bladder | 1,562 (2.2%) |
| Non-Hodgkin Lymphoma | 1,815 (2.6%) |
| Kidney | 827 (1.5%) |
| Leukemia | 822 (1.1%) |
| Thyroid | 1,587 (2.0%) |
| Other cancers | 33,512 (45.9%) |

^*N represents the unweighted number of survey respondents. Weighted percentages are reported.^

**Supplementary Table 2**. Unadjusted prevalence of health status, employment trends, and limitations among cancer survivors and general U.S. population.

| **Variable** | **Total (N=798,378)** | **U.S. population (N=725,691, 91.2%)** | **Cancer survivors (N=72,687, 8.8%)** |
| --- | --- | --- | --- |
| Any AL | 119,546 (15.6) | 99,113 (14.0) | 20,433 (33.9) |
| Any FL | 236,905 (33.7) | 202,187 (31.3) | 34,718 (61.1) |
| Need help with ADL | 15,146 (2.0) | 12,171 (1.7) | 2,975 (5.1) |
| Need help with IADL | 33,412 (4.1) | 26,968 (3.6) | 6,444 (10.0) |
| Need help with bath/shower | 10,337 (1.4) | 8,222 (1.2) | 2,115 (3.6) |
| Need help dressing | 8,558 (1.2) | 6,893 (1.0) | 1,665 (2.9) |
| Need help in/out of bed or chairs | 6,755 (0.9) | 5,444 (0.8) | 1,311 (2.3) |
| Need help eating | 3,237 (0.4) | 2,622 (0.4) | 615 (1.0) |
| Need help using the toilet | 4,738 (0.7) | 3,839 (0.6) | 899 (1.6) |
| Difficulty maintaining standing | 137,597 (19.1) | 115,109 (17.3) | 22,488 (40.2) |
| Difficulty maintaining sitting | 78,076 (10.9) | 67,061 (10.3) | 11,015 (19.1) |
| Difficulty walking | 45,361 (5.5) | 36,393 (4.8) | 8,968 (14.1) |
| Difficulty climbing stairs | 103,836 (13.4) | 85,260 (11.9) | 18,576 (29.9) |
| Difficulty relaxing | 28,954 (3.9) | 24,422 (3.6) | 4,532 (7.8) |
| Difficulty socializing | 54,287 (7.5) | 45,062 (6.7) | 9,225 (16.4) |
| Any work limitation | 123,831 (13.6) | 102,032 (12.4) | 21,799 (27.6) |
| Unable to work | 59,825 (6.1) | 49,827 (5.6) | 9,998 (12.3) |
| Work-lost days (mean) | 3.4 (11.1) | 3.2 (10.6) | 6.4 (18.0) |
| Health status, poor to fair | 114,747 (12.8) | 95,143 (11.6) | 19,604 (26.1) |

_*_ _All p-values are <0.001. N represents the unweighted number of survey respondents. Weighted percentages are reported._

**Supplementary Table 3**. Unadjusted prevalence of health status, employment trends, and limitations among cancer survivors and general U.S. population, stratified by marital status.

| **Variable** | **Married (N=361,460)** | **Living with partner (N=45,025)** | **Divorced/separated (N=132,676)** | **Widowed (N=80,594)** | **Never married (N=178,623)** |
| --- | --- | --- | --- | --- | --- |
| Any AL | 37,540 (12.3) | 4,155 (11.3) | 28,920 (25.1) | 28,211 (42.6) | 20,720 (12.4) |
| Any FL | 97,992 (33.0) | 10,272 (28.6) | 48,236 (43.4) | 44,569 (68.4) | 35,836 (21.7) |
| Need help with ADL | 4,046 (1.3) | 411 (1.1) | 3,145 (2.8) | 5,091 (8.7) | 2,453 (1.7) |
| Need help with IADL | 7,398 (2.4) | 752 (2.0) | 7,748 (6.6) | 11,982 (18.4) | 5,532 (3.5) |
| Need help with bath/shower | 2,689 (0.9) | 246 (0.6) | 1,865 (1.6) | 3,948 (6.8) | 1,589 (1.1) |
| Need help dressing | 2,582 (0.8) | 238 (0.6) | 1,513 (1.3) | 2,880 (5.1) | 1,345 (1.0) |
| Need help in/out of bed or chairs | 2,103 (0.7) | 227 (0.6) | 1,308 (1.2) | 2,110 (3.8) | 1,007 (0.7) |
| Need help eating | 797 (0.3) | 65 (0.2) | 538 (0.5) | 1,163 (2.0) | 674 (0.5) |
| Need help using the toilet | 1,456 (0.5) | 106 (0.2) | 760 (0.7) | 1,570 (2.9) | 846 (0.6) |
| Difficulty maintaining standing | 53,216 (17.9) | 5,153 (14.1) | 29,317 (26.5) | 30,495 (49.9) | 19,416 (11.1) |
| Difficulty maintaining sitting | 30,729 (10.3) | 3,775 (10.5) | 18,485 (16.6) | 13,168 (20.3) | 11,919 (7.0) |
| Difficulty walking | 12,096 (3.9) | 1,066 (2.8) | 10,680 (8.9) | 15,753 (24.1) | 5,766 (3.0) |
| Difficulty climbing stairs | 34,853 (11.3) | 3,420 (9.2) | 23,902 (20.9) | 28,167 (42.4) | 13,494 (7.3) |
| Difficulty relaxing | 9,915 (3.3) | 1,259 (3.5) | 7,209 (6.5) | 6,063 (9.7) | 4,508 (2.6) |
| Difficulty socializing | 17,722 (6.0) | 2,072 (5.8) | 12,764 (11.8) | 13,042 (22.0) | 8,687 (5.4) |
| Any work limitation | 39,711 (10.8) | 5,035 (11.0) | 30,773 (22.6) | 25,654 (31.7) | 22,658 (11.9) |
| Unable to work | 16,646 (4.5) | 2,244 (4.5) | 16,805 (11.8) | 12,732 (15.4) | 11,398 (5.3) |
| Work-lost days (mean) | 3.3 (11.1) | 3.9 (12.1) | 4.5 (13.5) | 4.2 (14.0) | 2.9 (9.3) |
| Health status, poor to fair | 41,763 (11.3) | 5,360 (11.5) | 27,096 (20.2) | 21,024 (27.0) | 19,504 (9.3) |

_*All p-values are <0.001._

**Supplementary Table 4**. Unadjusted prevalence of health status, employment trends, and limitations among cancer survivors, stratified by marital status.

| **Variable** | **Married (N=34,081)** | **Living with partner (N=2,280)** | **Divorced/separated (N=13,783)** | **Widowed (N=16,157)** | **Never married (N=6,386)** |
| --- | --- | --- | --- | --- | --- |
| Any AL | 7,216 (27.6) | 582 (33.3) | 4,600 (42.2) | 6,245 (50.8) | 1,790 (36.2) |
| Any FL | 14,722 (57.2) | 966 (55.7) | 6,837 (64.4) | 9,457 (77.6) | 2,736 (55.7) |
| Need help with ADL | 940 (3.6) | 76 (4.3) | 565 (5.4) | 1,179 (10.7) | 215 (4.9) |
| Need help with IADL | 1,628 (6.3) | 135 (7.1) | 1,414 (12.5) | 2,722 (22.2) | 545 (11.7) |
| Need help with bath/shower | 656 (2.5) | 54 (3.1) | 339 (3.2) | 918 (8.5) | 148 (3.2) |
| Need help dressing | 575 (2.1) | 48 (2.5) | 263 (2.5) | 660 (6.2) | 119 (2.5) |
| Need help in/out of bed or chairs | 470 (1.8) | 43 (2.3) | 241 (2.3) | 470 (4.5) | 87 (2.1) |
| Need help eating | 184 (0.7) | 15 (0.7) | 113 (1.0) | 252 (2.4) | 51 (1.1) |
| Need help using the toilet | 339 (1.3) | 21 (1.1) | 134 (1.3) | 340 (3.4) | 65 (1.3) |
| Difficulty maintaining standing | 8,992 (35.6) | 605 (35.0) | 4,485 (43.6) | 6,647 (59.2) | 1,759 (36.6) |
| Difficulty maintaining sitting | 4,332 (16.7) | 389 (22.1) | 2,690 (25.2) | 2,678 (22.3) | 926 (19.6) |
| Difficulty walking | 2,637 (10.0) | 186 (10.4) | 1,844 (16.3) | 3,598 (29.7) | 703 (13.8) |
| Difficulty climbing stairs | 6,495 (24.1) | 484 (26.3) | 3,881 (35.1) | 6,279 (49.6) | 1,437 (27.7) |
| Difficulty relaxing | 1,614 (6.2) | 171 (9.6) | 1,095 (10.4) | 1,271 (11.1) | 381 (8.1) |
| Difficulty socializing | 3,242 (13.0) | 263 (15.5) | 2,040 (20.9) | 2,883 (26.2) | 797 (17.5) |
| Any work limitation | 7,826 (22.4) | 694 (29.4) | 5,148 (36.8) | 6,108 (37.9) | 2,023 (31.3) |
| Unable to work | 3,161 (9.2) | 325 (13.0) | 2,698 (18.9) | 2,801 (17.3) | 1,013 (15.5) |
| Work-lost days (mean) | 5.8 (17.2) | 7.3 (19.2) | 7.4 (18.9) | 6.6 (19.8) | 7.8 (19.9) |
| Health status, poor to fair | 7,834 (22.8) | 685 (29.0) | 4,410 (32.7) | 4,851 (31.6) | 1,824 (28.6) |

_*All p-values are <0.001._

**Supplementary Table 5.** Multivariable Analysis of Outcomes Cancer Survivors compared to U.S. Population Using Survey-Weighted Models.

| **Variables (Ref: U.S. population)** | **AOR / β (95% CI)*** |
| --- | --- |
| Any AL | 1.44 (1.39 – 1.49) |
| Any FL | 1.61 (1.56 – 1.66) |
| Need help with ADL | 1.28 (1.20 – 1.36) |
| Need help with IADL | 1.28 (1.22 – 1.34) |
| Need help with bath/shower | 1.27 (1.18 – 1.38) |
| Need help dressing | 1.19 (1.09 – 1.29) |
| Need help in/out of bed or chairs | 1.25 (1.13 – 1.37) |
| Need help eating | 1.11 (0.96 – 1.28) |
| Need help using the toilet | 1.17 (1.04 – 1.31) |
| Difficulty maintaining standing | 1.48 (1.43 – 1.53) |
| Difficulty maintaining sitting | 1.26 (1.22 – 1.31) |
| Difficulty walking | 1.27 (1.22 – 1.32) |
| Difficulty climbing stairs | 1.38 (1.34 – 1.43) |
| Difficulty relaxing | 1.24 (1.18 – 1.31) |
| Difficulty socializing | 1.33 (1.27 – 1.38) |
| Any work limitation | 1.42 (1.37 – 1.47) |
| Unable to work | 1.29 (1.24 – 1.34) |
| Work-lost days (mean) | 2.71 (2.39 – 3.03) |
| Health status, poor to fair | 1.60 (1.54 – 1.65) |

_Abbreviations: AOR: adjusted odds ratio; AL: activity limitation;_ *_β_*_: beta-coefficient CI: confidence interval; FL: functional limitation.
*AOR and_ *_β_*_-coefficients represent estimates from survey-weighted regression models. Odds ratios (OR) are reported for binary outcomes and β-coefficients represent mean differences for continuous outcomes. All p-value <0.001._

**Supplementary Table 6**. Multivariable analysis of outcomes among cancer survivors only, using survey-weighted models with married survivors as the reference category.

| **Variables (Ref: Married survivors)** | **Living with partner** | **Divorced/separated** | **Widowed** | **Never married** |
| --- | --- | --- | --- | --- |
| Any AL | 1.50 (1.28 – 1.76) | 1.73 (1.60 – 1.86) | 1.56 (1.46 – 1.68) | 1.38 (1.24 – 1.54) |
| Any FL | 1.11 (0.95 – 1.29) | 1.13 (1.05 – 1.22) | 1.31 (1.21 – 1.41) | 0.91 (0.83 – 1.01) |
| Need help with ADL | 1.38 (1.01 – 1.87) | 1.15 (0.98 – 1.35) | 1.61 (1.40 – 1.85) | 1.09 (0.85 – 1.41) |
| Need help with IADL | 1.28 (0.99 – 1.66) | 1.72 (1.53 – 1.92) | 2.17 (1.96 – 2.40) | 1.61 (1.34 – 1.92) |
| Need help with bath/shower | 1.56 (1.10 – 2.21) | 0.95 (0.78 – 1.17) | 1.81 (1.54 – 2.13) | 1.02 (0.74 – 1.41) |
| Need help dressing | 1.39 (0.94 – 2.04) | 0.86 (0.69 – 1.08) | 1.60 (1.33 – 1.92) | 0.89 (0.62 – 1.28) |
| Need help in/out of bed or chairs | 1.40 (0.92 – 2.12) | 0.93 (0.74 – 1.17) | 1.35 (1.11 – 1.64) | 0.83 (0.53 – 1.28) |
| Need help eating | 1.36 (0.70 – 2.63) | 1.20 (0.86 – 1.69) | 1.70 (1.26 – 2.28) | 1.27 (0.67 – 2.41) |
| Need help using the toilet | 0.93 (0.50 – 1.73) | 0.79 (0.58 – 1.08) | 1.50 (1.18 – 1.92) | 0.66 (0.44 – 0.98) |
| Difficulty maintaining standing | 1.14 (0.98 – 1.33) | 1.20 (1.12 – 1.29) | 1.38 (1.29 – 1.49) | 0.98 (0.88 – 1.09) |
| Difficulty maintaining sitting | 1.25 (1.06 – 1.48) | 1.22 (1.13 – 1.33) | 0.96 (0.88 – 1.04) | 0.88 (0.78 – 1.00) |
| Difficulty walking | 1.47 (1.17 – 1.84) | 1.57 (1.43 – 1.73) | 2.12 (1.93 – 2.31) | 1.50 (1.29 – 1.75) |
| Difficulty climbing stairs | 1.29 (1.09 – 1.53) | 1.26 (1.16 – 1.36) | 1.52 (1.41 – 1.65) | 1.01 (0.90 – 1.15) |
| Difficulty relaxing | 1.35 (1.06 – 1.73) | 1.26 (1.12 – 1.42) | 1.17 (1.04 – 1.32) | 0.80 (0.65 – 0.98) |
| Difficulty socializing | 1.18 (0.97 – 1.45) | 1.36 (1.24 – 1.49) | 1.32 (1.20 – 1.44) | 1.04 (0.89 – 1.20) |
| Any work limitation | 1.38 (1.17 – 1.62) | 1.53 (1.42 – 1.65) | 1.23 (1.14 – 1.33) | 1.28 (1.15 – 1.44) |
| Unable to work | 1.34 (1.11 – 1.63) | 1.56 (1.43 – 1.71) | 1.20 (1.09 – 1.31) | 1.28 (1.12 – 1.46) |
| Work-lost days (mean) | 0.30 (-1.09 – 1.68) | 0.61 (-0.06 – 1.28) | 0.59 (-0.66 – 1.85) | 0.18 (-0.68 – 1.03) |
| Health status, poor to fair | 1.20 (1.03 – 1.41) | 1.21 (1.12 – 1.31) | 0.93 (0.86 – 1.01) | 0.92 (0.81 – 1.03) |

_Abbreviations: ADL, activities of daily living; AOR, adjusted odds ratio; AL, activity limitation; β, beta-coefficient; CI, confidence interval; FL, functional limitation; IADL, instrumental activities of daily living; ref, reference. *Models were restricted to respondents with a self-reported history of cancer and adjusted for age, sex, race, ethnicity, body mass index, household income, insurance type, employment status (except for the employment outcomes) and survey year. Odds ratios are reported for binary outcomes and β-coefficients represent adjusted mean differences for continuous outcomes._

**Supplementary Table 7.** NHIS Variables Used to Define Functional, Activity, and Employment-Related Outcomes

| **Outcome** | **NHIS variable** |
| --- | --- |
| Any activity limitation | LANY |
| Any functional limitation | FLANY |
| ADL limitation | LADL |
| IADL limitation | LAIADL |
| Bathing | LABATH |
| Dressing | LADRESS |
| Transferring (bed/chair) | LABED |
| Eating | LAEATH |
| Toileting | LATOILT |
| Standing for 2 hours | FLSTAND2HR |
| Sitting for 2 hours | FLSIT2HR |
| Walking | LAWALK |
| Climbing stairs | FLCLIMB |
| Leisure activities | FLRELAX |
| Socializing | FLSOCIAL |
| Self-rated health | HEALTH |
| Any work limitation / inability to work | LAMTWRK |
| Workdays lost due to illness | WLDAYR |
